# 1,2-Diphenyl-2-[(1-phenyl­eth­yl)amino]­ethanol

**DOI:** 10.1107/S1600536812027420

**Published:** 2012-06-27

**Authors:** Qing-Gao Hou, Chang-Qiu Zhao

**Affiliations:** a College of Chemistry and Chemical Engineering, Liaocheng University, Shandong 252059, People’s Republic of China

## Abstract

In the mol­ecule of the title compound, C_22_H_23_NO, there are two chiral atoms (R* for the C atom attached to the OH group and S* for the C atom attached to the phenyl ring). In the crystal, neighbouring mol­ecules are connected into a chain along the *b* axis by N—H⋯O hydrogen bonds.

## Related literature

For background to the synthesis of chiral organic compounds, see: Alcaide *et al.* (1981 ▶)
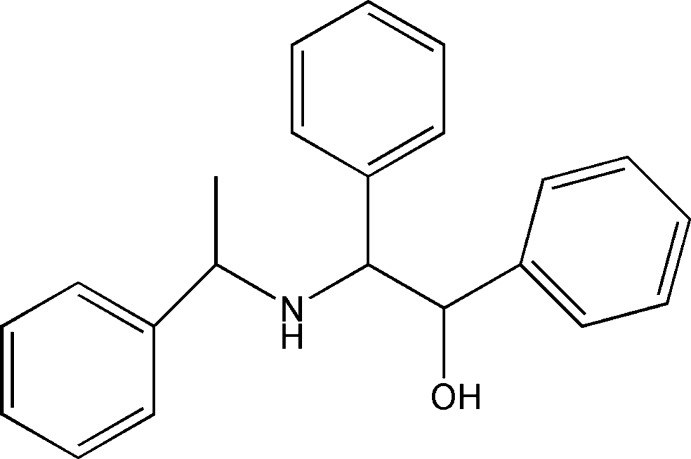


## Experimental

### 

#### Crystal data

C_22_H_23_NO
*M* *_r_* = 317.41
Orthorhombic, 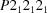

*a* = 6.307 (4) Å
*b* = 12.801 (7) Å
*c* = 22.490 (12) Å
*V* = 1815.7 (17) Å^3^
*Z* = 4
Mo *K*α radiation
μ = 0.07 mm^−1^
*T* = 298 K
0.50 × 0.29 × 0.21 mm

#### Data collection

Siemens SMART CCD area-detector diffractometer
Absorption correction: multi-scan (*SADABS*; Sheldrick, 1996 ▶) *T*_min_ = 0.966, *T*_max_ = 0.985
11619 measured reflections
4471 independent reflections
2660 reflections with *I* > 2σ(*I*)
*R*_int_ = 0.071

#### Refinement

*R*[*F* ^2^ > 2σ(*F* ^2^)] = 0.059
*wR*(*F* ^2^) = 0.160
*S* = 1.00
4471 reflections
218 parameters
H-atom parameters constrained
Δρ_max_ = 0.32 e Å^−3^
Δρ_min_ = −0.30 e Å^−3^

### 

Data collection: *SMART* (Siemens, 1996 ▶); cell refinement: *SAINT* (Siemens, 1996 ▶); data reduction: *SAINT*; program(s) used to solve structure: *SHELXS97* (Sheldrick, 2008 ▶); program(s) used to refine structure: *SHELXL97* (Sheldrick, 2008 ▶); molecular graphics: *SHELXTL* (Sheldrick, 2008 ▶); software used to prepare material for publication: *SHELXTL*.

## Supplementary Material

Crystal structure: contains datablock(s) I, global. DOI: 10.1107/S1600536812027420/ds2194sup1.cif

Structure factors: contains datablock(s) I. DOI: 10.1107/S1600536812027420/ds2194Isup2.hkl

Supplementary material file. DOI: 10.1107/S1600536812027420/ds2194Isup3.cml

Additional supplementary materials:  crystallographic information; 3D view; checkCIF report

## Figures and Tables

**Table 1:** Hydrogen-bond geometry (Å, °)

*D*—H⋯*A*	*D*—H	H⋯*A*	*D*⋯*A*	*D*—H⋯*A*
N1—H1*A*⋯O1^i^	0.90	2.04	2.908 (2)	160

Symmetry code: (i) 
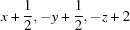
.

**Fig. 1.**
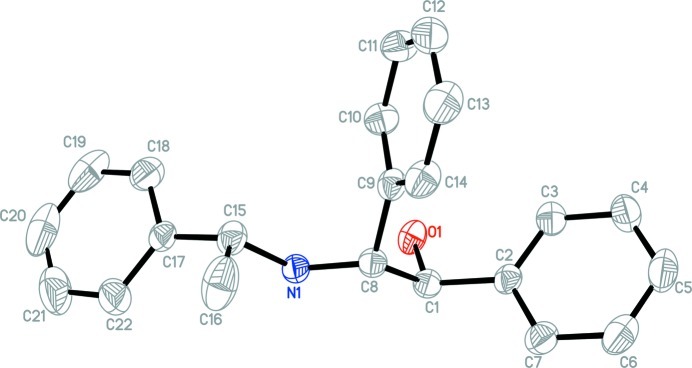
The molecular structure of the compound. Displacement ellipsoids are drawn at the 50% probability level.

**Fig. 2.**
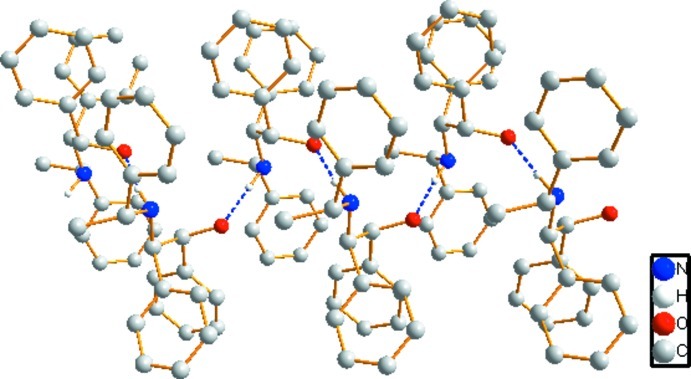
The one–dimensional chain, linked by N—H···O hydrogen bonds.

## supplementary crystallographic information

### Comment

In the molecule of chiral title compound 1,2-dipheny1-2-((1-phenylethyl)amino)ethanol,which derived from (R_p_) phenylamine,accroding to the Cram rules and the molecular structure, two chiral atoms: C1(R*) and C8(S*) are observed. In the crystal structure, intermolecular N—H···O hydrogen bonds connect the same neighbour molecules into a one-dimensional chain,giving rise along b axis. The angle of C1-O1-H1,O1-N1-H1A, C8-N1-H1A and C15-N1-H1A are 109.47°, 160.4°, 108.11° and 108.19°. The torsion angle of O1-C1-C8-N1 is 56.46 (0.22)°. The arrangement between two neighbour molecules are same.

### Experimental

Benzil (0.75 g, 3.6 mmol) was added to a stirred ethanol solution of *R*-phenylethylamine (0.87 g, 3.96 mmol) in a round-bottomed flask under a nitrogen atmosphere and heated until ethanol refluxed. This reaction took about 31 h. In the ice bath environment, sodium borohydride (0.27 g, 7.92 mmol) was added to the mixture in batches. Then drained ethanol, white solid was obtained. The crude product was extracted with dichloromethane three times. The organic phase was dried over anhydrous magnesium sulfate and then evaporated. The pure product was obtained after recrystallized with petroleum ether. Single crystal of the title compound suitable for *X*–ray diffraction were obtained by slow evaporation of petroleum ether solution of the title compound.

### Refinement

All H atoms attached to C N O atoms were fixed geometrically and treated as riding with C—H = 0.93 - 0.98 Å, N—H = 0.90 Å, O—H = 0.820 Å with *U*_iso_(H) = 1.5 *U*_eq_(methyl) and *U*_iso_(H) = 1.2 *U*_eq_(C) for all other H atoms.In the absence of significant anomalous scattering, Friedel pairs were merged and the absolute structure was assigned arbitrarily.

### Crystal data

**Table:**

C_22_H_23_NO	*F*(000) = 680
*M**_r_* = 317.41	*D*_x_ = 1.161 Mg m^−^^3^
Orthorhombic, *P*2_1_2_1_2_1_	Mo *K*α radiation, λ = 0.71073 Å
Hall symbol: P 2ac 2ab	Cell parameters from 3097 reflections
*a* = 6.307 (4) Å	θ = 2.4–21.5°
*b* = 12.801 (7) Å	µ = 0.07 mm^−^^1^
*c* = 22.490 (12) Å	*T* = 298 K
*V* = 1815.7 (17) Å^3^	Needle, colourless
*Z* = 4	0.50 × 0.29 × 0.21 mm

### Data collection

**Table:**

Siemens SMART CCD area-detector diffractometer	4471 independent reflections
Radiation source: fine-focus sealed tube	2660 reflections with *I* > 2σ(*I*)
Graphite monochromator	*R*_int_ = 0.071
phi and ω scans	θ_max_ = 28.3°, θ_min_ = 2.4°
Absorption correction: multi-scan (*SADABS*; Sheldrick, 1996)	*h* = −8→8
*T*_min_ = 0.966, *T*_max_ = 0.985	*k* = −16→17
11619 measured reflections	*l* = −29→22

### Refinement

**Table:**

Refinement on *F*^2^	Primary atom site location: structure-invariant direct methods
Least-squares matrix: full	Secondary atom site location: difference Fourier map
*R*[*F*^2^ > 2σ(*F*^2^)] = 0.059	Hydrogen site location: inferred from neighbouring sites
*wR*(*F*^2^) = 0.160	H-atom parameters constrained
*S* = 1.00	*w* = 1/[σ^2^(*F*_o_^2^) + (0.0777*P*)^2^] where *P* = (*F*_o_^2^ + 2*F*_c_^2^)/3
4471 reflections	(Δ/σ)_max_ = 0.001
218 parameters	Δρ_max_ = 0.32 e Å^−^^3^
0 restraints	Δρ_min_ = −0.30 e Å^−^^3^

### Special details

**Table:**

Geometry. All e.s.d.'s (except the e.s.d. in the dihedral angle between two l.s. planes) are estimated using the full covariance matrix. The cell e.s.d.'s are taken into account individually in the estimation of e.s.d.'s in distances, angles and torsion angles; correlations between e.s.d.'s in cell parameters are only used when they are defined by crystal symmetry. An approximate (isotropic) treatment of cell e.s.d.'s is used for estimating e.s.d.'s involving l.s. planes.
Refinement. Refinement of *F*^2^ against ALL reflections. The weighted *R*-factor *wR* and goodness of fit *S* are based on *F*^2^, conventional *R*-factors *R* are based on *F*, with *F* set to zero for negative *F*^2^. The threshold expression of *F*^2^ > σ(*F*^2^) is used only for calculating *R*-factors(gt) *etc*. and is not relevant to the choice of reflections for refinement. *R*-factors based on *F*^2^ are statistically about twice as large as those based on *F*, and *R*- factors based on ALL data will be even larger.

### Fractional atomic coordinates and isotropic or equivalent isotropic displacement parameters (Å^2^)

**Table:**

	*x*	*y*	*z*	*U*_iso_*/*U*_eq_	
N1	0.1361 (3)	0.17083 (12)	0.92509 (8)	0.0483 (5)	
H1A	0.2115	0.2278	0.9353	0.058*	
O1	−0.1676 (3)	0.15306 (12)	1.01455 (7)	0.0583 (5)	
H1	−0.1779	0.1634	0.9787	0.087*	
C1	0.0499 (4)	0.13193 (16)	1.02933 (10)	0.0485 (5)	
H1C	0.1173	0.1976	1.0414	0.058*	
C2	0.0702 (4)	0.05485 (16)	1.07981 (10)	0.0470 (5)	
C3	−0.0921 (5)	−0.01341 (18)	1.09504 (11)	0.0610 (7)	
H3	−0.2206	−0.0099	1.0749	0.073*	
C4	−0.0662 (6)	−0.0858 (2)	1.13920 (12)	0.0772 (8)	
H4	−0.1770	−0.1307	1.1487	0.093*	
C5	0.1249 (6)	−0.0928 (2)	1.17002 (12)	0.0811 (9)	
H5	0.1427	−0.1424	1.1998	0.097*	
C6	0.2869 (5)	−0.0255 (2)	1.15575 (12)	0.0775 (9)	
H6	0.4153	−0.0295	1.1760	0.093*	
C7	0.2594 (5)	0.04835 (19)	1.11128 (11)	0.0621 (7)	
H7	0.3693	0.0942	1.1024	0.075*	
C8	0.1619 (4)	0.09159 (15)	0.97284 (9)	0.0455 (5)	
H8	0.3136	0.0881	0.9820	0.055*	
C9	0.0926 (4)	−0.01903 (16)	0.95574 (9)	0.0478 (5)	
C10	−0.1051 (4)	−0.03981 (18)	0.93349 (11)	0.0580 (6)	
H10	−0.2015	0.0145	0.9285	0.070*	
C11	−0.1636 (5)	−0.1413 (2)	0.91822 (12)	0.0695 (7)	
H11	−0.2984	−0.1546	0.9032	0.083*	
C12	−0.0218 (6)	−0.2215 (2)	0.92537 (13)	0.0787 (9)	
H12	−0.0601	−0.2891	0.9148	0.094*	
C13	0.1766 (6)	−0.2023 (2)	0.94810 (11)	0.0790 (9)	
H13	0.2721	−0.2569	0.9535	0.095*	
C14	0.2331 (5)	−0.10146 (18)	0.96280 (10)	0.0621 (7)	
H14	0.3680	−0.0885	0.9777	0.075*	
C15	0.2056 (4)	0.13711 (19)	0.86494 (10)	0.0557 (6)	
H15	0.1306	0.0720	0.8558	0.067*	
C16	0.4411 (5)	0.1131 (3)	0.86352 (13)	0.0943 (11)	
H16A	0.5194	0.1738	0.8757	0.141*	
H16B	0.4818	0.0939	0.8239	0.141*	
H16C	0.4711	0.0563	0.8901	0.141*	
C17	0.1383 (4)	0.21679 (17)	0.81918 (9)	0.0529 (6)	
C18	−0.0514 (5)	0.2047 (2)	0.78936 (12)	0.0708 (7)	
H18	−0.1364	0.1473	0.7982	0.085*	
C19	−0.1189 (7)	0.2751 (3)	0.74684 (14)	0.1008 (12)	
H19	−0.2490	0.2665	0.7280	0.121*	
C20	0.0119 (11)	0.3595 (3)	0.73269 (16)	0.1194 (17)	
H20	−0.0280	0.4064	0.7031	0.143*	
C21	0.1949 (9)	0.3727 (3)	0.76188 (19)	0.1124 (15)	
H21	0.2794	0.4302	0.7530	0.135*	
C22	0.2613 (6)	0.3028 (2)	0.80495 (12)	0.0826 (9)	
H22	0.3894	0.3136	0.8245	0.099*	

### Atomic displacement parameters (Å^2^)

**Table:**

	*U*^11^	*U*^22^	*U*^33^	*U*^12^	*U*^13^	*U*^23^
N1	0.0643 (13)	0.0404 (8)	0.0402 (9)	−0.0054 (8)	0.0012 (9)	−0.0029 (8)
O1	0.0641 (12)	0.0634 (9)	0.0473 (8)	0.0145 (8)	−0.0044 (8)	0.0033 (7)
C1	0.0546 (14)	0.0432 (11)	0.0478 (12)	0.0012 (10)	−0.0004 (11)	−0.0010 (10)
C2	0.0614 (14)	0.0433 (10)	0.0363 (10)	0.0026 (10)	0.0002 (11)	−0.0068 (9)
C3	0.0647 (16)	0.0626 (14)	0.0556 (14)	−0.0012 (13)	0.0005 (13)	0.0061 (12)
C4	0.106 (2)	0.0668 (16)	0.0587 (16)	−0.0146 (16)	0.0050 (18)	0.0115 (14)
C5	0.125 (3)	0.0666 (16)	0.0515 (15)	0.0088 (19)	−0.0084 (19)	0.0114 (13)
C6	0.094 (2)	0.0794 (17)	0.0587 (16)	0.0127 (18)	−0.0215 (17)	−0.0033 (15)
C7	0.0732 (18)	0.0578 (13)	0.0552 (14)	−0.0007 (13)	−0.0096 (14)	−0.0035 (12)
C8	0.0490 (12)	0.0431 (10)	0.0446 (11)	0.0008 (10)	0.0004 (10)	−0.0011 (9)
C9	0.0598 (15)	0.0451 (11)	0.0385 (11)	0.0054 (10)	0.0038 (11)	−0.0021 (9)
C10	0.0578 (16)	0.0548 (13)	0.0613 (14)	0.0041 (11)	−0.0007 (13)	−0.0060 (11)
C11	0.0788 (19)	0.0655 (15)	0.0643 (15)	−0.0126 (14)	−0.0010 (15)	−0.0126 (13)
C12	0.128 (3)	0.0460 (13)	0.0621 (16)	−0.0081 (16)	0.0044 (18)	−0.0076 (13)
C13	0.120 (3)	0.0516 (14)	0.0654 (16)	0.0206 (17)	−0.0123 (18)	−0.0053 (13)
C14	0.0788 (18)	0.0537 (12)	0.0540 (13)	0.0154 (12)	−0.0063 (14)	−0.0070 (11)
C15	0.0649 (16)	0.0579 (12)	0.0443 (12)	0.0038 (12)	0.0067 (11)	−0.0047 (11)
C16	0.077 (2)	0.144 (3)	0.0618 (18)	0.038 (2)	0.0151 (16)	0.011 (2)
C17	0.0719 (17)	0.0502 (11)	0.0368 (11)	−0.0049 (12)	0.0050 (12)	−0.0075 (10)
C18	0.079 (2)	0.0720 (16)	0.0615 (15)	−0.0004 (15)	−0.0066 (15)	−0.0065 (15)
C19	0.130 (3)	0.107 (3)	0.0654 (19)	0.032 (2)	−0.031 (2)	−0.016 (2)
C20	0.214 (6)	0.091 (3)	0.053 (2)	0.040 (3)	0.006 (3)	0.0041 (19)
C21	0.183 (5)	0.0677 (19)	0.086 (2)	−0.012 (3)	0.035 (3)	0.0164 (19)
C22	0.116 (3)	0.0656 (15)	0.0658 (15)	−0.0227 (17)	0.0110 (17)	−0.0010 (14)

### Geometric parameters (Å, º)

**Table:**

N1—C8	1.486 (3)	C11—C12	1.371 (4)
N1—C15	1.486 (3)	C11—H11	0.9300
N1—H1A	0.9000	C12—C13	1.374 (4)
O1—C1	1.437 (3)	C12—H12	0.9300
O1—H1	0.8200	C13—C14	1.380 (4)
C1—C2	1.510 (3)	C13—H13	0.9300
C1—C8	1.543 (3)	C14—H14	0.9300
C1—H1C	0.9800	C15—C17	1.510 (3)
C2—C3	1.389 (4)	C15—C16	1.517 (4)
C2—C7	1.390 (4)	C15—H15	0.9800
C3—C4	1.368 (3)	C16—H16A	0.9600
C3—H3	0.9300	C16—H16B	0.9600
C4—C5	1.393 (5)	C16—H16C	0.9600
C4—H4	0.9300	C17—C18	1.380 (4)
C5—C6	1.375 (4)	C17—C22	1.384 (4)
C5—H5	0.9300	C18—C19	1.382 (4)
C6—C7	1.387 (4)	C18—H18	0.9300
C6—H6	0.9300	C19—C20	1.396 (6)
C7—H7	0.9300	C19—H19	0.9300
C8—C9	1.531 (3)	C20—C21	1.339 (6)
C8—H8	0.9800	C20—H20	0.9300
C9—C10	1.370 (4)	C21—C22	1.384 (5)
C9—C14	1.387 (3)	C21—H21	0.9300
C10—C11	1.394 (4)	C22—H22	0.9300
C10—H10	0.9300		
			
C8—N1—C15	115.29 (17)	C12—C11—H11	120.1
C8—N1—H1A	108.1	C10—C11—H11	120.1
C15—N1—H1A	108.1	C11—C12—C13	120.3 (3)
C1—O1—H1	109.5	C11—C12—H12	119.9
O1—C1—C2	112.21 (19)	C13—C12—H12	119.9
O1—C1—C8	108.02 (18)	C12—C13—C14	119.4 (3)
C2—C1—C8	111.21 (17)	C12—C13—H13	120.3
O1—C1—H1C	108.4	C14—C13—H13	120.3
C2—C1—H1C	108.4	C13—C14—C9	121.3 (3)
C8—C1—H1C	108.4	C13—C14—H14	119.3
C3—C2—C7	118.0 (2)	C9—C14—H14	119.3
C3—C2—C1	122.3 (2)	N1—C15—C17	109.95 (18)
C7—C2—C1	119.7 (2)	N1—C15—C16	111.5 (2)
C4—C3—C2	121.1 (3)	C17—C15—C16	113.5 (2)
C4—C3—H3	119.4	N1—C15—H15	107.2
C2—C3—H3	119.4	C17—C15—H15	107.2
C3—C4—C5	120.5 (3)	C16—C15—H15	107.2
C3—C4—H4	119.7	C15—C16—H16A	109.5
C5—C4—H4	119.7	C15—C16—H16B	109.5
C6—C5—C4	119.1 (3)	H16A—C16—H16B	109.5
C6—C5—H5	120.5	C15—C16—H16C	109.5
C4—C5—H5	120.5	H16A—C16—H16C	109.5
C5—C6—C7	120.2 (3)	H16B—C16—H16C	109.5
C5—C6—H6	119.9	C18—C17—C22	117.6 (3)
C7—C6—H6	119.9	C18—C17—C15	119.9 (2)
C6—C7—C2	121.0 (3)	C22—C17—C15	122.5 (3)
C6—C7—H7	119.5	C17—C18—C19	122.0 (3)
C2—C7—H7	119.5	C17—C18—H18	119.0
N1—C8—C9	114.75 (17)	C19—C18—H18	119.0
N1—C8—C1	108.46 (16)	C18—C19—C20	118.8 (4)
C9—C8—C1	112.69 (18)	C18—C19—H19	120.6
N1—C8—H8	106.8	C20—C19—H19	120.6
C9—C8—H8	106.8	C21—C20—C19	119.7 (4)
C1—C8—H8	106.8	C21—C20—H20	120.2
C10—C9—C14	118.4 (2)	C19—C20—H20	120.2
C10—C9—C8	122.1 (2)	C20—C21—C22	121.5 (4)
C14—C9—C8	119.5 (2)	C20—C21—H21	119.2
C9—C10—C11	120.8 (2)	C22—C21—H21	119.2
C9—C10—H10	119.6	C21—C22—C17	120.4 (4)
C11—C10—H10	119.6	C21—C22—H22	119.8
C12—C11—C10	119.8 (3)	C17—C22—H22	119.8
			
O1—C1—C2—C3	21.1 (3)	C14—C9—C10—C11	−0.1 (4)
C8—C1—C2—C3	−100.0 (3)	C8—C9—C10—C11	179.9 (2)
O1—C1—C2—C7	−161.18 (19)	C9—C10—C11—C12	−0.1 (4)
C8—C1—C2—C7	77.7 (3)	C10—C11—C12—C13	0.7 (4)
C7—C2—C3—C4	−0.8 (3)	C11—C12—C13—C14	−1.0 (4)
C1—C2—C3—C4	177.0 (2)	C12—C13—C14—C9	0.8 (4)
C2—C3—C4—C5	−0.1 (4)	C10—C9—C14—C13	−0.2 (4)
C3—C4—C5—C6	0.4 (4)	C8—C9—C14—C13	179.8 (2)
C4—C5—C6—C7	0.1 (4)	C8—N1—C15—C17	170.64 (18)
C5—C6—C7—C2	−1.0 (4)	C8—N1—C15—C16	−62.6 (3)
C3—C2—C7—C6	1.3 (3)	N1—C15—C17—C18	−93.4 (2)
C1—C2—C7—C6	−176.5 (2)	C16—C15—C17—C18	140.9 (3)
C15—N1—C8—C9	−43.9 (3)	N1—C15—C17—C22	87.2 (3)
C15—N1—C8—C1	−170.87 (19)	C16—C15—C17—C22	−38.4 (3)
O1—C1—C8—N1	56.5 (2)	C22—C17—C18—C19	−0.2 (4)
C2—C1—C8—N1	−179.96 (17)	C15—C17—C18—C19	−179.5 (2)
O1—C1—C8—C9	−71.7 (2)	C17—C18—C19—C20	1.7 (5)
C2—C1—C8—C9	51.9 (3)	C18—C19—C20—C21	−2.5 (5)
N1—C8—C9—C10	−53.9 (3)	C19—C20—C21—C22	1.8 (6)
C1—C8—C9—C10	70.9 (3)	C20—C21—C22—C17	−0.2 (5)
N1—C8—C9—C14	126.1 (2)	C18—C17—C22—C21	−0.6 (4)
C1—C8—C9—C14	−109.1 (2)	C15—C17—C22—C21	178.8 (3)

### Hydrogen-bond geometry (Å, º)

**Table:**

*D*—H···*A*	*D*—H	H···*A*	*D*···*A*	*D*—H···*A*
N1—H1*A*···O1^i^	0.90	2.04	2.908 (2)	160

Symmetry code: (i) *x*+1/2, −*y*+1/2, −*z*+2.
